# Men’s experiences of radiotherapy treatment for localized prostate cancer and its long-term treatment side effects: a longitudinal qualitative study

**DOI:** 10.1007/s10552-020-01380-3

**Published:** 2021-01-04

**Authors:** E. Sutton, J. A. Lane, M. Davis, E. I. Walsh, D. E. Neal, F. C. Hamdy, M. Mason, J. Staffurth, R. M. Martin, C. Metcalfe, T. J. Peters, J. L. Donovan, J. Wade, The ProtecT Study Group, P. Holding, J. W. F. Catto, D. J. Rosario, H. Kynaston, O. Hughes, P. Bollina, A. Doherty, V. Gnanapragasam, R Kockelbergh, A Paul, E. Paez, D. Gillatt, E Rowe, J. Oxley

**Affiliations:** 1 Department of Population Health Sciences, Bristol Medical School, University of Bristol, Canynge Hall, Bristol, UK; 2 Bristol Randomised Trials Collaboration, Population Health Sciences, Bristol Medical School, Canynge Hall, 39 Whatley Road, Clifton, Bristol, BS8 2PS UK; 3 National Institute for Health Research Collaboration for Leadership in Applied Health Research and Care (NIHR CLAHRC) West, University Hospitals Bristol NHS Trust, Bristol, UK; 4 Nuffield Department of Surgical Sciences, University of Oxford, Oxford, UK; 5 Division of Cancer & Genetics, School of Medicine, Cardiff University, Cardiff, UK; 6 Department of Oncology, Cardiff University, Cardiff, UK

**Keywords:** Prostate cancer, Radiotherapy, External-beam radiotherapy, Treatment experiences, Treatment side effects, Qualitative research

## Abstract

**Purpose:**

To investigate men’s experiences of receiving external-beam radiotherapy (EBRT) with neoadjuvant Androgen Deprivation Therapy (ADT) for localized prostate cancer (LPCa) in the ProtecT trial.

**Methods:**

A longitudinal qualitative interview study was embedded in the ProtecT RCT. Sixteen men with clinically LPCa who underwent EBRT in ProtecT were purposively sampled to include a range of socio-demographic and clinical characteristics. They participated in serial in-depth qualitative interviews for up to 8 years post-treatment, exploring experiences of treatment and its side effects over time.

**Results:**

Men experienced bowel, sexual, and urinary side effects, mostly in the short term but some persisted and were bothersome. Most men downplayed the impacts, voicing expectations of age-related decline, and normalizing these changes. There was some reticence to seek help, with men prioritizing their relationships and overall health and well-being over returning to pretreatment levels of function. Some unmet needs with regard to information about treatment schedules and side effects were reported, particularly among men with continuing functional symptoms.

**Conclusions:**

These findings reinforce the importance of providing universal clear, concise, and timely information and supportive resources in the short term, and more targeted and detailed information and care in the longer term to maintain and improve treatment experiences for men undergoing EBRT.

**Supplementary Information:**

The online version of this article (10.1007/s10552-020-01380-3) contains supplementary material, which is available to authorized users.

## Introduction

Prostate cancer (PCa) is the most common cancer in males in the UK with 47,151 cases diagnosed in 2015 [1]. Randomized controlled trials (RCTs) evaluating treatment options for clinically localized PCa (LPCa) have most often evaluated radical surgery and conservative management with frequent monitoring of the disease (e.g., active surveillance, active monitoring) [2, 3]. However, men may also receive external-beam radiotherapy (EBRT). Observational studies have shown that EBRT and brachytherapy have concomitant risks of bowel, sexual, and urinary dysfunction [4, 5]. The prostate testing for cancer and treatment (ProtecT) RCT compared radical prostatectomy (RP), EBRT with neoadjuvant androgen deprivation therapy (ADT), and active monitoring (AM) [6] and found very high survival rates and no evidence of differences in PCa or all-cause mortality between treatment groups after a median 10-year follow-up [6]. Patient-reported outcome measures (PROMs) showed that men allocated to EBRT reported higher levels of bowel dysfunction than the other groups, with erectile function affected particularly at six months. Urinary voiding and nocturia were worse in the EBRT group at 6 months, but mostly recovered after 12 months and were then similar to other groups [7]. Quality of life related to functional symptoms mirrored these effects [7]. In this paper, we describe an investigation using qualitative methods undertaken to explore in detail individual men’s perspectives and experiences of the EBRT treatment pathway.

Some previous qualitative studies have produced combined findings for men receiving RP or EBRT, making it difficult to distinguish specific EBRT experiences [8–10]. Previous studies specifically focusing on EBRT [11–13] followed up men between 6 weeks and one year only, finding unmet information needs regarding treatment options and side effects [11, 13], and some men feeling unsafe [13] or concerned about their ability to keep a full bladder during treatment [12]. The studies reported that many developed close relationships with the healthcare team because of the intensity and duration of treatment [11]. Men also reported ‘normalization’ of sexual functioning within the context of growing older [12, 13].

The ProtecT qualitative study investigated men’s experiences of receiving EBRT with neoadjuvant ADT for newly diagnosed LPCa from baseline up to eight years of follow-up. The aims were to explore men’s experiences of treatment and its side effects over time, to provide contextual insights alongside the trial’s PROMs [7].

## Subjects/patients (or materials) and methods

### ProtecT study

The ProtecT RCT methods have been described in detail [6, 14, 15]. Men declining randomization were offered identical follow-up and formed a comprehensive cohort within the study design [14]. ProtecT EBRT comprised neoadjuvant ADT for a minimum of three months and maximum of six months before and during 3-D conformal EBRT delivered at a dose of 74 Gy in 37 fractions [15]. This involved receiving hormone pellets injected into the stomach and attending a local oncology center every weekday for seven weeks. Men were asked to attend with a moderately full bladder and to empty the rectum of feces and flatus if possible. The analysis reported here was undertaken after 8 years of follow-up was completed to provide a longitudinal perspective.

### Interview study

In-depth qualitative interviews explored men’s experiences of treatment and related side effects [16]. The sampling strategy for the qualitative interviews is described in detail elsewhere [15]. Sampling included men who were invited to take part in the interview study following diagnosis and decision-making about participation in the RCT, with serial interviews undertaken in a longitudinal design to explore men’s experiences over time [18]. Maximum variation sampling was used to include men from each treatment arm, those who accepted randomization or chose a treatment, older and younger men, a range of socioeconomic backgrounds, those with lower and higher-risk cancer, from four of the nine study centers. This sample was augmented by a small number of men recruited prior to diagnosis and interviewed at points during the diagnosis process then at three yearly intervals following treatment [15]. Most interviews were conducted by JW; others by JD, LB, and LS (see acknowledgments) using the interview schedule in Table 1. Interviews were conducted face-to-face in the man’s home, hospital outpatient department, or university premises, or by telephone and were audio-recorded. The first interviews of all but one of the men whose data are reported in the present study took place from 4 to 11 months post-EBRT (mean 8 months); subsequent interviews took place at around 2- to 3-year intervals facilitating exploration of perceived treatment outcomes and the impact of side effects over time. Interview timings for individual men differed to take account of their treatment schedules and availability, with additional interviews if the research team became aware of major changes in the man’s treatment or disease. One man was recruited pre-diagnosis [15] and was interviewed both pre- and post-EBRT (Table 2).

**Table 1:** Interview topics

*Introductions* (including interviewer’s professional background, purpose, and length of interview and explanation of how confidentiality will be maintained)
• Decision-making whether to participate in the ProtecT study
• Experiences of the diagnostic process
• Impact of prostate cancer diagnosis at the time of diagnosis and ongoing
• Decision-making whether to accept random allocation to a treatment or to choose a treatment
• Views on radical treatments
• Expectations of HT/RT—including information provision
• Views and experiences of HT/RT to date
Impact of HT/RT
Any changes/improvements to RT treatment?
Experiences of ProtecT study participation
*Second and third interviews explored*
Experience of HT/RT since last contact, positive and negative
Ongoing impact of HT/RT
Reflections on treatment decision-making and any changes in views with time/events
Expectations of treatment vs outcomes
Experiences of living with PCa
Experiences of ProtecT study participation

**Table 2:** Men’s age at first interview and number of interviews

Randomized to RT (Pseudonym)	Marital status	Age at first interview	No of interviews
Ralph	M	60–64	3
Derek	M	55–59	3
Stephen	M	65–69	3
Ray	M	55–59	3
Len	M	55–59	3
Alex	M	65–69	3
Terrence	M	65–69	3
Bernard	M	60–64	3
Clive	D	60–64	3
Donald	M	64–69	3
Ned	M	60–64	3
*Chose RT*			
Ivor	M	64–69	3
Geoff	M	64–69	3
Keith	M	60–64	2 (Deceased)
Alan	M	60–64	3
Henry	M	60–64	8 (3 post-RT)

*M* married, *D* Divorced

Written consent was obtained and participants were informed that they could withdraw from the study at any time. If men requested their partner to be present, they were invited to complete the same consent process. Four men included their partners in their first interview, but no partners were present for the following interviews. Interview recordings and field notes were reviewed by researchers conducting the interviews in order to develop follow-up interview questions, and analysis was carried out after all the data had been collected. Interviews were transcribed verbatim and data were analyzed thematically [19, 20] with the aid of NVivo software by ES and JW who met regularly to discuss the themes emerging from the data, comparing coding for a sample of transcripts, modifying the coding framework, and developing recurring themes. Case studies for each participant were developed to enable us to track changes over time and explore how men adapted to their symptoms. A descriptive account was written up encompassing the interview themes. The researchers actively sought and discussed deviant cases to challenge the development of themes. Multicenter research ethics committee approval was obtained from the UK East Midlands (formerly Trent) Multicenter Research Ethics Committee (01/4/025).

## Results

Twenty-three men in the EBRT group were invited to participate in qualitative interviews and 16 agreed to take part (3 did not respond, 3 refused, and 1 excluded as he did not have a telephone). Eleven had been randomized and 5 chose EBRT as their preferred treatment (Table 3). Most were interviewed at least three times at key points during treatment. Fourteen men participated in 3 interviews, 1 in 2 interviews (deceased), and 1 in 8 interviews (3 post-EBRT). Fifteen of the men were married at the time of the first interview and one divorced. Four main themes emerged: experiences of treatment, experiences and impact of treatment side effects, perceptions of outcome, and reflections on information and support needs. Each theme is reported below with quotations from men assigned pseudonyms to protect their identities. Additional quotations are presented in a Supplementary Table. Age at time of interview and length of time post-EBRT provide contextual information.

**Table 3:** Characteristics of interview participants

Study participants *n*=16		
	Accepted random allocation	Chose treatment
	*n*=11	*n*=5
Age at diagnosis		
50–54 years	1	0
55–59 years	2	1
60–64 years	5	2
65–69 years	3	2
Social class		
Managerial/professional	6	1
Other	5	4
Study center		
1	0	1
2	3	1
3	5	2
4	3	1
Grade and stage		
Gleason 6, T1	7	3
Higher grade or stage	4	2

## Experiences of treatment

Most men indicated that the EBRT pathway proceeded well and as expected. Several experienced temporary discomfort when receiving hormone injections, and the need to have a full bladder caused some anxiety:

The [hormone] injection was painful, but it’s bearable … after a half hour, pain was gone. (Alan, 63, 6m post RT)

One of the difficult things was having to sort of inflate your bladder with drinking a lot before the actual session … that was fine if everything ran and generally it did … but if things overran then here you were bloated with all this liquid and dying to go to the loo. (Henry, 62, 1y 10m post RT)

Many anticipated that 35 sessions of EBRT over 7 weeks would be arduous, but most were surprised at how easily it passed:

Thirty odd times I’ve had to go to there. Awful long time, but once you start doing it and you’re ticking off the days, you know, the weeks. It soon goes. (Ralph, 70, 5y 10m post RT)

I was going to sleep on the bed, it was that relaxing. It didn’t feel as though I was fighting cancer. (Bernard, 66, 5y 6m post RT)

## Experiences and impact of the side effects of EBRT/ADT

All men reported experiencing some bowel, sexual, or urinary side effects. Symptoms attributed to ADT included hot flushes, weight gain, breast enlargement and tiredness, some of which caused embarrassment:

They started me off on the hormone treatment in September … I’m still getting hot flushes … my wife laughs every time, because she says now you know what it’s like [laughs]. (Terrence, 67, 8m post RT)

I noticed the breast enlargement a bit as well, and that’s quite a bit … shocking … you think good God … what’s happening here? (Clive, 64, 4m post RT)

Problems with fatigue and disrupted sleep occurred and for some persisted, with men attributing this to various reasons including travel to hospital for treatment, age or comorbidities:

I’m perhaps slightly more tired than I was but that could be an age process anyway … I seem to sleep a lot … but it may be an element of age creeping on rather than the radiotherapy. (Henry, 62, 1y 10m post RT)

I also find now that I seem to be permanently tired. I don’t know whether that’s the anti- depressants because they can have that effect, or maybe it’s my approach to life. (Bernard, 66, 5y 6m post RT)

Most men reported bowel problems including urgency, loose motions/diarrhea, passing bloody mucus, or needing to strain, which, for some, persisted:

About 6 or 7 weeks … I was having problems with desperate sort of urgency and having to rush to the toilet. (Ned, 61, 6m post RT)

My toilet habits in the morning are a bit of a strain. I need to use the toilet three, sometimes four times of a morning before my day starts. (Derek, 62, 6y post RT)

Two men were diagnosed with radiation proctitis (inflammation and damage to the colon after radiation exposure), causing severe and persisting problems:

I had a lot … a fair bit of pain and bleeding at one time … I sort of started getting over that and then I started getting some heavy bleeding from the back passage. (Stephen, 69, 2y 7m post RT)

It sort of flares up a couple of times a month … I get a bit of bolting pain in the stomach and I pass a mucus sort of deposit … and then it goes away and settles. (Alex, 70, 5y 4m post RT)

Urinary side effects were mostly not severe and reported at the first interview (4-12m post RT), including voiding, increased frequency, and some pain, although a few men experienced continuing issues, with nocturia contributing to fatigue:

There was a slight sort of stinging sensation for a while … it was there when you went you could sort of feel it, it was sort of acidic um sort of sensation. (Terrence, 76, 8m post RT)

It’s very rare I have a night without having to get up. Usually only once, but sometimes it might be, you know, two or three times. (Ned, 64, 2y 11m post RT)

However, only three men sought prescribed medication for urinary symptoms:

I went to the doctors and that helped a little bit just to realise that I wasn’t suffering on my own… it was just getting ridiculous. (Bernard, 66, 5y 6m post RT)

Most coped with urinary and bowel symptoms by being careful about what they drank, accommodating changes or being aware of the location of toilet facilities:

I decided that the stodge diet - bread, starchy foods - would be better than the healthy … five a day. (Ivor, 69, 7m post RT)

So that’s the way I sort of plan it, my day… around the toilet. (Bernard, 63, 2y 9m post RT)

Difficulties with sexual function were common, with adverse impacts including lack of “*sex drive*” or desire, inability to maintain an erection or achieve a climax:

I don’t know with the radiotherapy, but the hormone treatment definitely affected my sex life [laughs]… instead of being ready and wanting to do it … it just takes it out of your mind almost. You’ve really got to say, you know, I haven’t had anything this week … almost force yourself to do it. (Terrence, 67, 8m post RT)

For some, there was recovery, but more reported anxiety about its lack:

There was some problems with [sexual intercourse] during and after the treatment that’s sort of regularised itself now after two years. (Donald, 71, 2y 9m post RT)

I have been waiting and waiting and waiting thinking it will come back, it will come back, it will come back and unfortunately it has not, or it does not appear to be coming back. I just wish I could get it fixed … why is it not gone back? Everything else appears to have gone back to the way it was. (Clive, 65, I yr, 8 mths post RT)

Treatments offered by clinical staff were helpful for some, but not used by others:

I did have trouble with my erection, I went onto tablets but after so long everything has come right again. (Len, 60, 5y post RT)

I was offered Viagra. I think at one consultation with [urologist] he did give me a prescription for it, which I took to the dispensary and had tablets and I put them in a drawer and they’ve never been used. (Derek, 57, 9m post RT)

Men delayed or rejected treatment or help-seeking for various reasons, including having a strong relationship, family health issues, caring responsibilities, or waiting until they felt physically stronger:

We’ve been married fifty years and it’s not a problem for me and my wife … we’re happy as we are. (Ralph, 70, 5y 10m post RT)

And like the nurse said, if we get any problems, just ring her and she’ll put a prescription down for me … but at the moment, you know I think my body’s had enough so give it a twelve month now give it a rest and see how it comes back then like. (Keith, 61, 11m post RT)

For those whose problems persisted in the longer term, it often became difficult to distinguish between the impact of comorbidities, normal aging, and treatment side effects. Many men expected that their sexual activity would decrease as they aged and general health became more important:

The most important thing for me is that is that I feel quite healthy so [the] sexual side of it is not over burdening because I’ve been married for over 30 years … I probably would be more worried if I was in my 30s. (Ray, 61, 5y 5m post RT)

It doesn’t bother me very much because it’s more, it’s more of a sort of macho thing than anything else. It’s not that I’m in any sort of relationship or sort of looking for any relationship it was more a case of you know what I mean, you feel old [laughs]. (Clive, 68, 4y 6m post RT)

## Perceptions of outcome

Some men worried about follow-up test results and whether treatment had been successful:

The only question I keep asking myself is that, is it a cure? … I would like to think that I have been cured but what extent would it be, where I could say yes I’m cured, that’s the question… I would probably say at this present time that I’m living with it and with a few issues. (Ray, 61, 5y 5m post RT)

If you have the operation and have the prostate removed and that sort of thing then that’s done and dusted …. [with EBRT] I think it’s one these things where you can never be absolutely 100% certain that it’s gone. (Donald, 73, 4y 10m post RT)

In contrast, others considered their treatment complete, particularly if they experienced few side effects, with some comparing their experience to others they perceived as less fortunate:

When you’re told, you know, it’s a bit of a shock but … after you’ve had the treatment no problem at all. I was working up until the last week … I was getting very tired … I had a week off and then you’re back to normal. (Keith, 62, 1y 9m post RT)

I don’t feel as though I’ve had prostate cancer, compared to the stories that you hear in the newspapers, friends and stuff like that. I still don’t feel as though I’ve had the full-blown thing. It’s like having a cold compared to pneumonia. (Bernard, 63, 2y 9m post RT)

## Reflections on information/support needs

During interviews, participants reflected on the study information sheet detailing potential side effects of treatment and leaflets provided by the local hospital. There were some aspects of timing and details of treatments they felt could be improved:

I think we waited three maybe four months for the radiotherapy to start, which whilst it wasn’t the end of the world, I wasn’t in pain or feeling ill, it did sort of mess my life up a bit if you like. Shall we go on holiday? ….. These [hormone] implants have to take time to work. That much wasn’t explained. (Derek, 57, 9m post RT)

Lots of clicks and bangs. OK, I know that all the machinery makes a lot of noise … some people might appreciate the technical advice and other people, it might frighten them away. (Geoff, 67, 9 m post RT)

Those who experienced more or serious side effects felt they had not been sufficiently prepared – suggesting that information should be provided on an ongoing basis (not just at the start) to men who do experience such side effects:

I didn’t know very much about radiotherapy and the side-effects (Ned, 61, 6m post RT)

They could sit you down and talk to you more about what is going on, … what is likely to happen to you. (Stephen, 72, 4y 2m post RT) [And]

They tell you that there can be side-effects but … I wasn’t aware that was something you could get [radiation proctitis] as bad as I did get (Stephen, 73, 5y 7m post RT)

However, many men described in positive terms the support and information they had received from healthcare professionals and members of the ProtecT team:

I think the thing that really stands out is the help and support actually in the oncology through all my treatment. I think that was just absolutely outstanding … It wasn’t one of these things, “*Oh God, I’ve gotta go have radiotherapy tomorrow*.” It was, I know it sounds ridiculous, but it was a pleasure to go there. Now that in itself was as good as the treatment. (Donald, 73, 4y 10 m post RT)

Another important factor was the support they received from peers, friends, and family:

[A] group got together [at hospital] and started talking with one another … the best part of it was the fact that the group of people who were there, they weren’t frightened to talk to one another … it was good fun … eased the pressure off of everybody. (Alex, 65, 11m post RT)

Every time I went to [hospital] [wife] was with me … we used to make a little thing we’d either go down in the morning perhaps stop for lunch on the way back or do a little bit of shopping or something like that, and or friends would come with us so we used to make a bit of a day out. (Ralph, 65, 9m post RT)

## Discussion

The findings reported here illustrate the ways that men organized their lives post-EBRT, developing coping strategies to accommodate side effects that continued in the longer term and by getting on with their lives. For many, their narratives enabled post-EBRT changes to be normalized and blurred together with expected age-related changes. They described the support they had received from clinical teams in glowing terms. However, there were some men who continued to worry about the outcome of their treatment or struggled with longer-term problems, such as radiation proctitis, or persisting issues with sexual function, for whom more information and care were needed.

These qualitative findings align with evidence from quantitative PROMS [7] but provide important additional contextual and experiential insights into men’s recovery. Men experienced a range of side effects on bowel, sexual, and urinary function as well as hot flushes, weight gain, and fatigue attributed to ADT. They generally reported satisfaction with the care received, particularly valuing the support provided by health professionals and members of the ProtecT research team over the lengthy treatment period: a factor noted in previous studies [11, 21].

Men reported their experiences of the adverse impact of the side effects of EBRT and ADT on urinary, bowel, and sexual function. However, in contrast to the findings of previous research [8], rather than solely referring to age in relation to a loss of sexual function, the men in our study demonstrated adaptive preferences when discussing all these areas of impact, finding it difficult to unpick the effects of aging, comorbidities, and related treatment from the impact of EBRT or ADT. As with other studies, a few men experienced difficulties in retaining a full bladder during EBRT treatment and temporary pain from hormone injections [12], but unlike Oster et al’s study [13], ProtecT men did not report feeling unsafe. A small number of men reported embarrassment with breast enlargement, and although some referred to the impact on their masculine identity [22], none reported directly avoiding intimacy [23, 24] and they were generally accepting of their post-treatment persona [13].

Men tended to downplay the impacts of treatment generally and there was reticence to seek help, particularly with regard to sexual function [10, 21] but also for urinary problems. Some exhibited temporalizing—waiting to see if things improved before seeking help [25]. Factors affecting decisions to seek help included partner influence, family health issues, caring responsibilities, and needing time to recover. Many men emphasized the importance of having a strong relationship with their partner and prioritized recovery from PCa over returning to pretreatment levels of sexual function [9, 10, 12]. None of the men reported accessing psychosexual services but were provided with the opportunity to discuss sexual function problems with study nurses. Appropriate timing of information giving has been highlighted in previous research [26]. Some men in this study reported unmet needs with regard to information on treatment schedules and side effects of ADT and EBRT [21], particularly when they experienced more severe or longer-term symptoms. Men also highlighted the difficulties of interpreting follow-up test results and wanting to know whether their treatment was successful [10, 21], with uncertainty impacting upon anxiety levels and coping capacity. Other men reported that life had reverted back to “*normal*” even while coping with persisting treatment impacts, aligning with narratives of a “*new normal*” reported by Baker et al. [27].

The importance of support from others echoed findings from previous studies [11, 13, 19, 24, 28], as did strategies of coping with bowel and urinary symptoms by changing fluid intake or being aware of the location of toilet facilities [13, 29]. A few of the men also made downward comparisons referring to others they believed were less fortunate [30].

In comparison to the study PROMS where urinary function was function was mostly recovered at 12 months, a few men in our study reported longer-term urinary symptoms. It may be that the interviews gave the men greater opportunities to reflect on longer-term side effects in more detail. We found no notable differences in the experiences of men who were younger (50–59) or older (60–69) at diagnosis, or for those who were randomized to, or chose EBRT, or between those with low (Gleason 6) or intermediate (Gleason 7+) risk cancer.

Men in the randomized group were slightly more likely to report anxiety regarding the success of their treatment, although we recognize that, due to the modest number of men interviewed, in statistical terms, this may not reflect a difference in the study population as a whole.

This study has both strengths and limitations. Limitations of the study include that none of the study participants were from minority ethnic groups or identified themselves as gay or bisexual. The majority were married so we recognize that perceptions regarding body image and sexual function might differ amongst single men. A key strength is the data collection embedded within the ProtecT trial and three interviews conducted with most participants over 8 years of follow-up. This clearly contrasts with previous research with a maximum of 12 months of follow-up [12] and usually less [11, 13]. A total of 47 interviews were conducted post-RT allowing in-depth exploration of individual issues and providing evidence of how men’s narratives evolved over time.

## Conclusions

This study has provided an in-depth insight into men’s experiences of EBRT and neoadjuvant ADT, and the strategies employed to cope with these over the longer term, which add to the quantitative findings from the ProtecT trial. Some important gaps in information provision and support were found, particularly for men experiencing severe or longer-term symptoms. Many of the symptoms relating to treatment side effects improved over time, although normalization and adaptation were evident in many accounts, leading to deferral or failure to seek help. This needs to be considered when designing service and information provision, particularly with regard to impact on sexual function and related treatments—regardless of patient age. The importance of support from health professionals, family, and peers was clear, but some men expressed anxiety regarding follow-up tests and not knowing if their treatment had been successful. These findings highlight the importance of providing clear, concise, and timely information and supportive resources in the short term, and more targeted and detailed information and care in the longer term to maintain and improve treatment experiences for men undergoing EBRT.

## Supplementary Information

Below is the link to the electronic supplementary material.

Supplementary material 1 (DOCX 22 kb)
