# Supplementary material for: Men’s experiences of radiotherapy treatment for localized prostate cancer and its long-term treatment side effects: a longitudinal qualitative study
Source: Cancer Causes Control. 2021 Jan 4;32(3):261–9. doi: 10.1007/s10552-020-01380-3 (PMC7870600; doi:10.1007/s10552-020-01380-3)
Supplement: Supplementary file 1 — Supplementary material 1 (DOCX 22 kb) [file 10552_2020_1380_MOESM1_ESM.docx]

**Supplementary Table: Additional participant quotations**

| **Experiences of treatment** | |
| --- | --- |
| Satisfaction with care | I got nothing but praise for that system I really haven’t … everyone I spoke to I’ve told them it’s marvellous … and like I say if I’d have paid thousands of pounds out wouldn’t have had better service. (Ralph, 65, 9m post RT) |
| Length of treatment | You actually think, am I ever gonna get through this? You think it’s, crikey, go back and fore, you know, all this time. (Len, 60, 5y post RT) |
| **Experiences and impact of the side-effects of EBRT/ADT** | |
| Fatigue | I think it was the travelling back and forth more than anything with the radiotherapy. (Len, 55, 9m post RT) |
| Impact on bowel function – short and longer term | There was a lot more mucus content, for want of a better description … it wasn’t the diarrhoea that you’d have if you’d eaten something that you shouldn’t, you know, that was off or something like that. (Clive, 64, 4m post RT)  Yeah, gets embarrassing now doesn't it? My toilet habits in the morning are a bit of a strain. I need to use the toilet three, sometimes four times of a morning before my day starts. (Derek, 62, 6y post RT) |
| Impact on urinary function –  short and longer term | Urinating was a problem … when you want to urinate you had to go straight away. You’re always a bit frightened of being too far from a toilet. (Alan, 63, 6m post RT)  I still have a slight tendency to be very urgent with my peeing … nothing that I can't deal with … it's just getting used to it. (Donald, 73, 4y 10m post RT) |
| Coping strategies - urinary and bowel symptoms | My wife bought me … a wee bottle you know and I had that in the car … one day I come home from the hospital I actually wet myself which is not nice, but I just couldn’t do nothing about it. (Ralph, 65, 9m post RT)  I control what I drink … later in the evening … after 6 o’clock. (Alex, 67, 2y 9m post RT)  I only used pads … if I was going to be on a trip … where I’d have to do something for a length of time where it could have got embarrassing. Just going down to the shops or going to the supermarket I rarely wore them. (Stephen, 72, 4y 2m post RT) |
| Normalization - ageing processes and urinary and bowel symptoms | It’s not as if I have to jump in a car and drive 50 miles up the motorway … my work life sort of accommodates it … on a scale of one to ten of inconvenience I suppose four, perhaps five. (Derek, 62, 6y post RT)  I think it’s just age related as far as I’m concerned and possibly there’s been a slight increase since the treatment … but nothing that it’s not manageable. (Donald, 71, 2y 9m post RT) |
| Impact on sexual function – short and longer term | The implants [ADT], they reduce the ability, they also reduce the desire (Derek, 57, 9m post RT)  I probably need to ask a question now, how much …regarding that because I, sexual side of it I feel, I feel I am, not quite right back, back to normal (Ray, 56, 8m post RT)  There was some problems with [sexual intercourse] during and after the treatment that’s sort of regularised itself now after two years. (Donald, 71, 2y 9m post RT) |
| Impact on sexual function - delay in help-seeking | But [nurse] did recommend something like a pump, an injection … I have a meeting with her [nurse] in January actually, so there is no great rush. So I shall probably talk to her in January about it. It isn’t causing me big issues at the moment. (Alan, 64, 2y 5m post RT) |
| Factors impacting on help-seeking – relationships and age | We are very happily married and yeah, we are the best of friends … the physical side, whereas it has always been enjoyable it has never been the be all and end all of our married life. (Derek, 57, 9m post RT)  [wife’s name] is happy with the state of things that we are at the moment … it’s something that hasn’t bothered [name] … so we’ll leave it. (Ivor, 71, 2y 8m post RT) |
| Normalization – ageing and decline in sexual activity | I’m getting older and all you know. Sort of wearing out a bit. (Geoff, 67, 9m post RT)  Well I think there has been one problem; the sex element … probably subconsciously I was maybe more aware that I had prostate cancer and what I was having to go through than maybe was on the surface and it was just afterwards, as things relaxed maybe that I was more aware. But it's not been a problem. I imagine it would have been different If I'd been younger. (Bernard, 66, 5y 6m post RT) |
| **Perceptions of Outcome** | |
| Anxiety about recurrence | I got anxious actually going to have it [PSA test] done anyway … waiting for the results you get used to … what’s it gonna be? You’re waiting for that word nought. (Len, 57, 2y 9m post RT)  Having been treated but I still see myself as living with prostate cancer. Until someone can come along and say for definite it’s obviously gone, I have not got it, I have to accept that I still have a form of prostate cancer and to be honest I don’t expect it to ever go away. (Terrence, 68, 3 yrs post RT)  that |
| **Reflections on information/support needs** | |
| Unmet information needs | They tell you that there can be side-effects but not … I wasn't aware that was something you could get [radiation proctitis] as bad as I did get (Stephen, 73, 5y 7m post RT)  The itching came as a surprise actually because I’d never … I didn’t have any sort of suggestion beforehand that it would happen you know … I wasn’t connecting it to the treatment (Clive, 64, 4m post RT) |
| Importance of support | The amount of support you get and the phone calls you get from people you haven’t seen for a long time … they phone you up to find out how you, you know, what’s going on. And they drag you out some of them. (Stephen, 68, 9m post RT) |
